# A modified transparent cap-assisted delivery method for capsule endoscopy

**DOI:** 10.1055/a-2299-2351

**Published:** 2024-04-29

**Authors:** Yao Yi, Ting-Ting Cao, Tao Gan, Zhu Wang

**Affiliations:** 1 Department of Gastroenterology and Hepatology, West China Hospital, Sichuan University, Chengdu, China; 2 Sichuan University-Oxford University Huaxi Joint Centre for Gastrointestinal Cancer, Chengdu, China

Capsule endoscopy stands as the primary diagnosis method for small bowel observation. However, in patients with gastroparesis, pyloric stenosis, and altered anatomy, capsules may lodge in the esophagus or stomach after ingestion. For such instances of capsule stagnation, direct endoscopic deployment should be performed. Various accessories for this include the endoscopic retrieval net and the AdvanCE capsule delivery device (US Endoscopy, Mentor, Ohio, USA) 1 2 , but due to considerations of accessibility and cost, the polypectomy snares designed are frequently chosen in clinical settings. The standard practice involves using a snare to grasp the capsule and orient the capsule transversely. This orientation can pose difficulties in navigating narrow passageways like the esophageal inlet and pylorus.

A modified method is proposed to address this limitation. The approach requires a snare (SAS-1-S; Cook Medical, Bloomington, Indiana, USA ), a transparent cap (D-201-10704; Olympus, Tokyo, Japan), and surgical suture thread or dental floss ( Fig. 1 ). Initially, the transparent cap is positioned on the tip of the scope as usual, ensuring alignment of the cap’s side hole with the instrument channel of the scope ( Fig. 2 ). The suture is threaded through this hole and tied at the tip of the snare, which is inserted from the instrument channel ( Fig. 3 ). Experimentally, adjusting the capsuleʼs orientation into the longitudinal direction by pulling the suture has been shown to simplify insertion ( Fig. 4 , Fig. 5 ). Clinically, this longitudinal orientation, achieved via the cap and snare, facilitates smoother passage through the pylorus compared to the conventional transverse orientation. Supporting video evidence demonstrates the efficacy of this method ( Video 1 ).

**Fig. 1:**
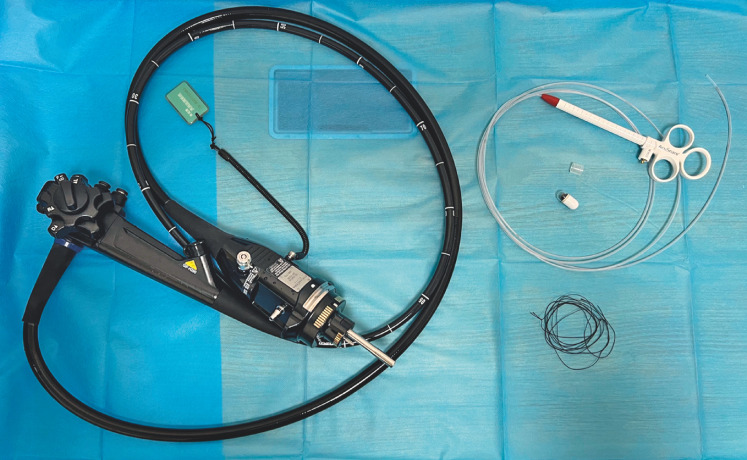
The modified method requires a snare, a transparent cap, and a surgical suture.

**Fig. 2:**
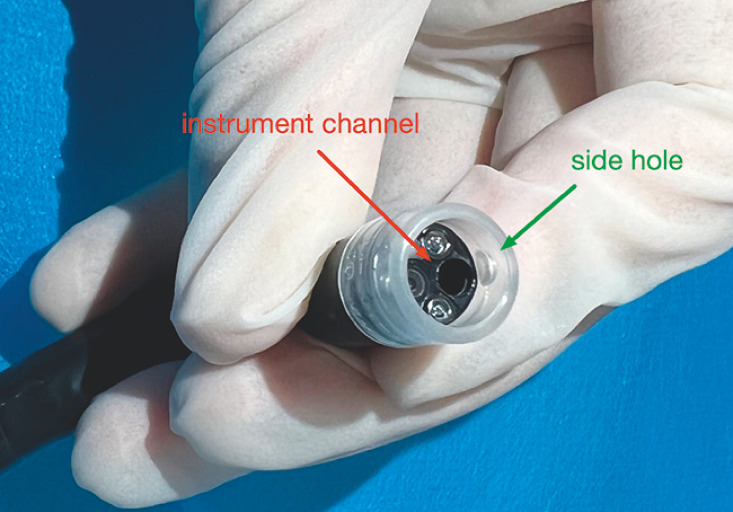
The side hole (green arrow) of the transparent cap needs to be placed in line with the instrument channel (red arrow).

**Fig. 3:**
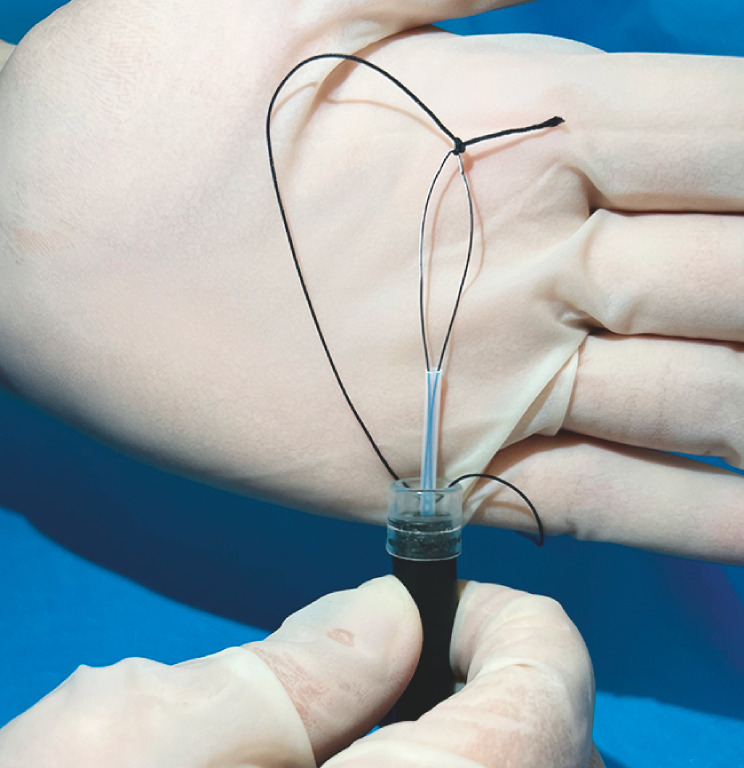
The surgical suture is threaded through the side hole of the transparent cap and tied at the tip of the snare.

**Fig. 4:**
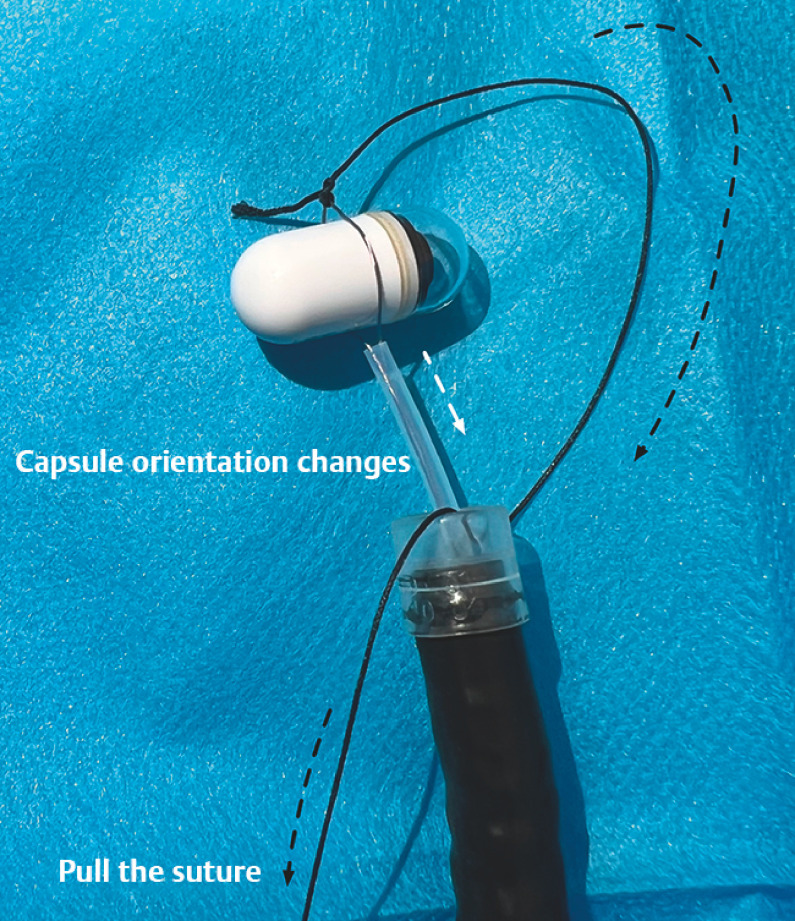
The snare grasps the capsule and positions it transversely.

**Fig. 5:**
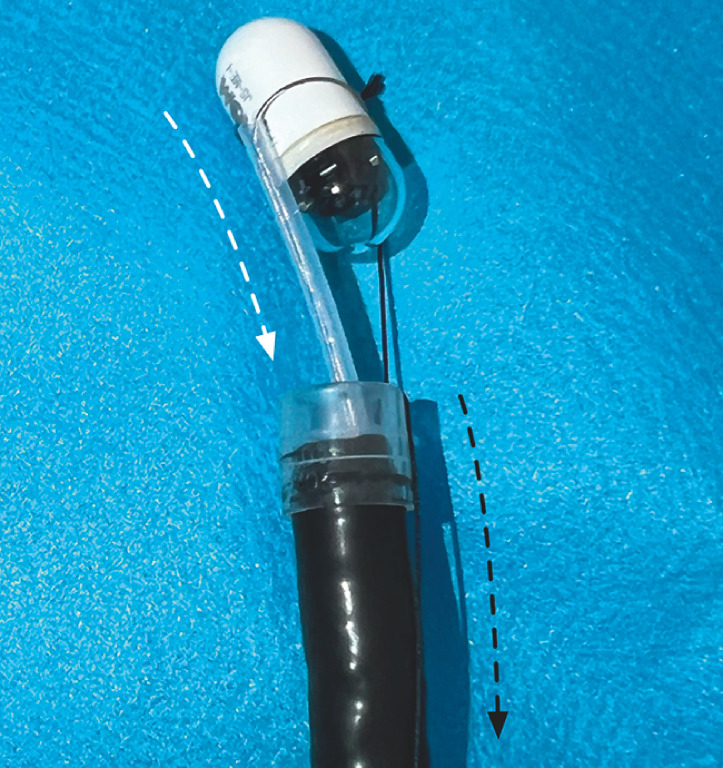
Pulling the suture (black arrow) changes the orientation of the capsule to the longitudinal direction (white arrow).

A modified transparent cap-assisted delivery method for capsule endoscopy.Video 1

In summary, this modified approach using a transparent cap may improve capsule delivery via a polypectomy snare, offering a practical and efficient solution to a common challenge in capsule endoscopy.

Endoscopy_UCTN_Code_TTT_1AO_2AL

## Notes

Endoscopy E-Videos https://eref.thieme.de/e-videos
*E-Videos* is an open access online section of the journal *Endoscopy* , reporting on interesting cases and new techniques in gastroenterological endoscopy. All papers include a high-quality video and are published with a Creative Commons CC-BY license. Endoscopy E-Videos qualify for HINARI discounts and waivers and eligibility is automatically checked during the submission process. We grant 100% waivers to articles whose corresponding authors are based in Group A countries and 50% waivers to those who are based in Group B countries as classified by Research4Life (see: https://www.research4life.org/access/eligibility/ ). This section has its own submission website at https://mc.manuscriptcentral.com/e-videos .

## References

[1] Sumioka A Oka S Tsuboi A Endoscopic delivery method using a retrieval net for patients with small-bowel capsule endoscopy stagnation in the stomach Gastroenterol Res Pract 2021 2021 3.216193E6 10.1155/2021/3216193 PMC8702352 34956361

[2] Ohmiya N Oka S Nakayama Y Safety and efficacy of the endoscopic delivery of capsule endoscopes in adult and pediatric patients: Multicenter Japanese study (AdvanCE-J study) Dig Endosc 2022 34 543 552 10.1111/den.14104 34379849

